# *Toxoplasma gondii* Infection in Kyrgyzstan: Seroprevalence, Risk Factor Analysis, and Estimate of Congenital and AIDS-Related Toxoplasmosis

**DOI:** 10.1371/journal.pntd.0002043

**Published:** 2013-02-07

**Authors:** Gulnara Minbaeva, Alexander Schweiger, Aigerim Bodosheva, Omurbek Kuttubaev, Adrian B. Hehl, Isabelle Tanner, Iskender Ziadinov, Paul R. Torgerson, Peter Deplazes

**Affiliations:** 1 State Sanitary Epidemiological Department of the Kyrgyz Republic, Bishkek, Kyrgyzstan; 2 Institute of Parasitology, University of Zurich, Zurich, Switzerland; 3 Department of Biology, Kyrgyz State Medical Academy, Bishkek, Kyrgyzstan; 4 Section of Epidemiology, Vetsuisse Faculty, University of Zurich, Zurich, Switzerland; University of Chicago, United States of America

## Abstract

**Background:**

HIV-prevalence, as well as incidence of zoonotic parasitic diseases like cystic echinococcosis, has increased in the Kyrgyz Republic due to fundamental socio-economic changes after the breakdown of the Soviet Union. The possible impact on morbidity and mortality caused by *Toxoplasma gondii* infection in congenital toxoplasmosis or as an opportunistic infection in the emerging AIDS pandemic has not been reported from Kyrgyzstan.

**Methodology/Principal Findings:**

We screened 1,061 rural and 899 urban people to determine the seroprevalence of *T. gondii* infection in 2 representative but epidemiologically distinct populations in Kyrgyzstan. The rural population was from a typical agricultural district where sheep husbandry is a major occupation. The urban population was selected in collaboration with several diagnostic laboratories in Bishkek, the largest city in Kyrgyzstan. We designed a questionnaire that was used on all rural subjects so a risk-factor analysis could be undertaken. The samples from the urban population were anonymous and only data with regard to age and gender was available. Estimates of putative cases of congenital and AIDS-related toxoplasmosis in the whole country were made from the results of the serology. Specific antibodies (IgG) against Triton X-100 extracted antigens of *T. gondii* tachyzoites from *in vitro* cultures were determined by ELISA. Overall seroprevalence of infection with *T. gondii* in people living in rural vs. urban areas was 6.2% (95%CI: 4.8–7.8) (adjusted seroprevalence based on census figures 5.1%, 95% CI 3.9–6.5), and 19.0% (95%CI: 16.5–21.7) (adjusted 16.4%, 95% CI 14.1–19.3), respectively, without significant gender-specific differences. The seroprevalence increased with age. Independently low social status increased the risk of *Toxoplasma* seropositivity while increasing numbers of sheep owned decreased the risk of seropositivity. Water supply, consumption of unpasteurized milk products or undercooked meat, as well as cat ownership, had no significant influence on the risk for seropositivity.

**Conclusions:**

We present a first seroprevalence analysis for human *T. gondii* infection in the Kyrgyz Republic. Based on these data we estimate that 173 (95% CI 136–216) Kyrgyz children will be born annually to mothers who seroconverted to toxoplasmosis during pregnancy. In addition, between 350 and 1,000 HIV-infected persons are currently estimated to be seropositive for toxoplasmosis. Taken together, this suggests a substantial impact of congenital and AIDS-related symptomatic toxoplasmosis on morbidity and mortality in Kyrgyzstan.

## Author Summary

A serological study on toxoplasmosis was undertaken in a rural and urban population in Kyrgyzstan. The observed seroprevalence was adjusted because of differences between age and gender stratifications in the study group compared to population census figures. This gave an estimated seroprevalence in rural and urban populations of 5.1% and 16.4% respectively. In our analysis we determined the risk-factors for infection in the rural population to be age, low social-status and low number of sheep owned. While the seroprevalence in this rural population was relatively low, the seroprevalence found in the urban population of Bishkek correlated better with international data. Extrapolating from our data, about 173 seroconversions during pregnancy may be expected annually in Kyrgyzstan. In addition, considering a prevalence of HIV-Toxoplasma-co-infection between 7/100,000 (official HIV-prevalence data) and 19.4/100,000 (UNAIDS-estimates), 350–1,000 people are at risk for AIDS-related toxoplasmosis. Therefore, in the face of the rising prevalence of HIV infection education of medical personnel on treatment and prevention of toxoplasmosis is recommended.

## Introduction

As the Central Asian countries became independent after the breakdown of the Soviet Union in 1991 fundamental socio-economic changes took place. This led, *inter alia*, to a deterioration of public infrastructure especially in the veterinary and public health sector. There is growing concern about the emergence of several neglected tropical diseases with a potentially high disease burden in these newly independent states [1]. Whilst epidemiological data show a rising incidence of some zoonotic diseases such as echinococcosis during the last twenty years [2] , there is currently no information about the prevalence of others, including toxoplasmosis. In general, there is a paucity of published literature on *Toxoplasma* seroprevalence in countries of the former Soviet Union both during and after the Soviet era. One study from the Russian city of Omsk suggests that the seroprevalence of toxoplasmosis has increased in post Soviet Russia [3].

In the Kyrgyz Republic there has been considerable migration to urban centres (mainly Bishkek and Osh) since 1991. Nevertheless, 66% of the population lives in rural areas (3.5/5.3 million people) and approximately 25% of the current urban population has recently moved in from rural areas (source: National Statistical Office of the Kyrgyz Republic (www.stat.kg)). In rural areas small subsistence type farming rather than large scale collectivised farming systems (Kolkhozes) has become common, which led to a considerable lowering of living standards for many people [4], [5]. Whereas risk factors for infection with *T. gondii* in urban centres are likely to be comparable to other urban areas around the world, in the rural areas life according to older pre- or early-Soviet era pastoralist traditions has emerged, representing a special epidemiological risk environment. In the neighbouring country of Uzbekistan, the seroprevalence ranged from 14.6% to 24.6% in individuals aged 15–40 years [6]. In comparison, seroprevalence of *T. gondii* infection in Europe ranges from 5–10% in the northern parts of Scandinavia [7] to 54% in Southern Europe. In the United States, data from a nationwide survey of subjects sampled between 1999 and 2004 suggested that the overall age adjusted *T. gondii* seroprevalence among persons of age 6–49 years was 10.8% [8]. In a region of Kazakhstan, socio-culturally similar to rural Kyrgyzstan the seroprevalence was determined at 16.1% [5].

The number of HIV-infected patients in the Kyrgyz Republic is growing rapidly: the estimated prevalence has risen tenfold from 0.02% in 2001 to 0.2% in 2009 [9]. Moreover, coverage with the highly active antiretroviral treatment (HAART) is low: approximately 1’900 patients (range: <1’000–2’700) are estimated to be in need of HAART, but only 231 are reported to receive this treatment (a coverage of 12%) [9], which puts the considerable proportion of dually infected patients at risk for developing AIDS-related toxoplasmosis.

Vertical transmission of the parasite in the case of a primary infection during pregnancy can lead to congenital toxoplasmosis with severe consequences and even to abortion [10]. Congenital toxoplasmosis is associated with a variety of syndromes including chorioretinitis and neurological problems such as hydrocephalus.

The aims of the present study were to measure the seroprevalence of *Toxoplasma* infection in two populations in Kyrgyzstan (rural and urban) as a basis for estimating the overall burden of toxoplasmosis in the Kyrgyz Republic. This includes calculating the incidence of congenital toxoplasmosis and estimating morbidity rates due to HIV-associated toxoplasmosis. In addition, a risk factor analysis was carried out in the rural study population.

## Materials and Methods

### Ethics statement

This cross-sectional study has received prior ethical approval by the Ministry of Health of the Kyrgyz Republic (Nr. 2008/258 and 2009/268) and was undertaken in the Kochkor district of Naryn Oblast in central Kyrgyzstan.

### The study area

Rural study area: The Kochkor district is located in a mountainous region in the central part of Kyrgyzstan in Naryn Oblast (province), and has a population of approximately 60’000. Livestock husbandry is the principal occupation of the local inhabitants (source: national statistical office of the Kyrgyz Republic (www.stat.kg)). The district comprises 11 counties with 31 villages. The study was conducted in 8 villages located in this district. This area was believed to be typical of many of the rural areas of Kyrgyzstan that have problems with echinococcosis. A concomitant surveillance study of echinococcosis was planned and this provided an opportunity to investigate toxoplamosis in a rural Kyrgyz population using the same study resources.

Urban study area: Bishkek is the capital and largest city of the Kyrgyz Republic with a population of approximately 900’000, and is surrounded by farmland (mainly cultivation of vegetables).

### Study-populations

Subjects were invited to participate in the rural study. Out of a total population of 9’914 in the Kochkor population, 1’065 volunteered to participate representing 10.7% of the target population. Blood samples were taken and all volunteers were asked to complete a questionnaire. Of the 1065 participants, four failed to provide the necessary data and were excluded.

A number of diagnostic laboratories were asked if they could supply anonymous random serological samples from blood that had been referred for routine diagnostic testing from city residents greater than 1 year of age. A total of 899 sera were supplied whose patient origin was Bishkek. Otherwise only the age and gender of the patients was known. This represented approximately 0.1% of the total city population.

### Questionnaire

The questionnaire included general information about the person, village of residence, name, age, sex, family size, nationality, occupation, and living standard, questions on livestock raised and specific facts which might constitute risk factors. These include the source of drinking water, cat ownership and number of cats owned, as well as eating habits: consumption of shashlyk, a Kyrgyz speciality made mainly from sheep meat, as the most likely source of undercooked meat, and consumption of home-made sour cream as the most likely source of unpasteurized milk, respectively. All questionnaires had unique numerical identifiers. To ensure the cultural appropriateness of the questions and to guarantee that each question was fully understood, the questionnaire was designed and tested for its comprehensibility by several of the authors with language skills in Russian, Kyrgyz and English.

### Serological test design and analysis

We developed and used a new test system rather than using the available commercial test kit partly for reasons of cost but more importantly as part of a technology transfer programme between Switzerland and Kyrgyzstan. The detailed methodology for this test is described in the Supplementary information S1.

### Statistical analysis

Data was collated in Excel (Microsoft Co., Redmond, WA) and imported into R (www.r-project.org/) for analysis. A mixed logistic modelling approach was implemented, with the various risk factors entered as fixed effects and the village of origin as a mixed effect. Risk factors with a p>0.15 value were sequentially removed from the model. In addition, the final random effects model was compared to a fixed effect model by quasi-likelihood to identify the most parsimonious model. The linearity of the increase in age-specific prevalence was tested using a Generalized Additive Model (GAM). For statistical evaluation of binomial data, the χ-square test with 95% confidence intervals according to Clopper and Pearson were used.

### Incidence of congenital and AIDS-related toxoplasmosis

The data we obtained in this study enabled us to estimate the likely incidence of congenital and AIDS related toxoplasmosis in Kyrgyzstan. The details of the methodology we used is given in Supplementary information S2 and S3.

## Results

### Characteristics of participants

Of the 1’061 participants from the rural Kochkor district and of the 899 patient sera from laboratories and hospitals in Bishkek, 728 (69%) and 611 (68%), respectively, were from women. The median age at the time of sampling in the Kochkor area was 34 years (interquartile range: 22–47) in women, and 27 years (interquartile range 12–45) in men, whereas in the urban population the median age at time of sampling was 30 years (interquartile range: 20–47) and 25 years (interquartile range: 7–44), respectively. The specifics of both populations participating in the study are summarised in table 1.

**Table 1:** Overview of gender and age groups in both study groups.

	Bishkek (N = 899)	Kochkor (N = 1061)	all (N = 1960)
Age (95%CI)	32.7 (31.5–33.9)	33.5 (32.4–34.6)	33.1 (32.3–33.9)
Gender			
female	611 (68%)	728 (69%)	1339 (68%)
male	288 (32%)	333 (31%)	621 (32%)

Age groups†				Proportion of each age group by census data
0–4 y*	46 (5%) (m = 24)	14 (1%)	60 (3%)	10.5%m = 5.45%
5–14 y*	120 (13%) (m = 64)	180 (17%)	300 (15%)	19.4%m = 10.1%
15–17 y*	19 (2%) (m = 6)	67 (6%)	86 (5%)	6.8%m = 3.5%
18–19 y	29 (3%) (m = 5)	26 (2%)	55 (3%)	4.6%m = 2.3%
20–24 y*	115 (13%) (m = 27)	100 (9%)	215 (11%)	10.7%m = 5.5%
25–29 y*	125 (14%) (m = 29)	98 (9%)	223 (11%)	8.7%m = 4.5%
30–34 y	89 (10%) (m = 26)	94 (9%)	183 (9%)	7.0%m = 3.7%
35–39 y	62 (7%) (m = 16)	89 (8%)	151 (8%)	5.8m = 2.9%
>40 y*	294 (33%) (m = 91)	393 (37%)	687 (35%)	26.7%m = 12.1%

* Significant differences,

† given by the data on age-specific HIV-prevalence from the National Centre for AIDS of the Kyrgyz Republic.

m = males (number of cases or proportion of cases).

### Seroprevalence

**Figure 1:**
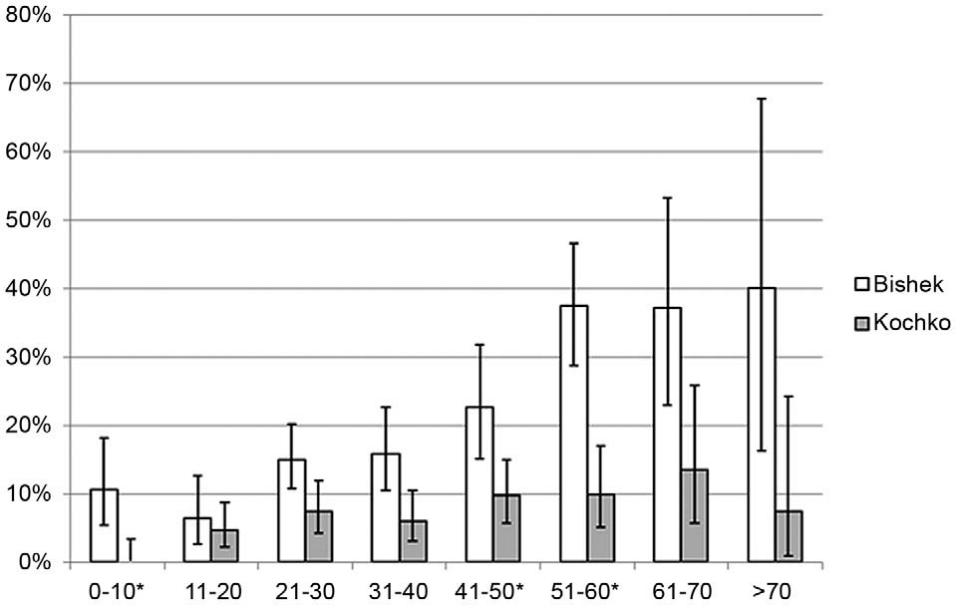
Age-specific seroprevalences of *T. gondii* infection. Seroprevalences according to decade of life in the rural Kochkor area (light grey) and Bishkek (white). Error bars indicate 95%CI. * Significant differences in age-specific seroprevalence.

The overall crude seroprevalence in the rural population was 6.2% (95%CI: 4.8–7.8) compared with 19.0% (95%CI: 16.5–21.7) in the urban population of Bishkek. When adjusted for the differences in age and gender profile of the sampled population compared to the census figures this indicated that the actual seroprevalence was 5.1% (95% CI 3.9–6.5 ) in the rural areas and 16.4% (95% CI 14.1–19.3) in the urban areas. The proportions of seropositive men from rural and urban areas were 5.7% (95%CI: 3.5–8.7) (adjusted 5.5%, 95% CI 3.0–7.8) and 18.4% (95%CI: 14.1–23.4), (adjusted 15.8% 95%CI, 12.0–20.8%) and of seropositive women 6.5% (95%CI: 4.8–8.5) (adjusted 4.6%, CI 3.5%–5.5%) and 19.3% (95%CI: 16.3–22.7) (adjusted 16.7%,95% CI = 13.0–19.4), respectively. The age- specific seroprevalences in both study groups are shown in figure 1. Neither significant gender differences in age-specific *T. gondii* seroprevalence, nor significant deviations from linear increase of the age-specific incidences were found. The calculated odds ratio for seropositivity per year of age in the Kochkor district was 0. 16 (95%CI: 0.10–0.22) for women, 0.19 (95%CI: 0.10–0.28) for men, and overall 0.185 (95%CI: 0.13–0.24); in urban Bishkek the corresponding calculated odds ratios were 0.59 (95%CI: 0.42–0.75), 0.88 (95%CI: 0.44–1.31), and overall 0.64 (95%CI: 0.49–0.79).

### Risk factor analysis

The particulars of the rural study population with respect to putative risk factors are summarized in table 2. Of those, only age, poor living standard and number of sheep owned were found to be statistically significant independent risk factors. The results of the risk-factor analysis are shown in table 3.

**Table 2:** Population characteristics and occurrence of putative risk factors in the Kochkor area according to serological result.

	Seronegative (N = 996)	Seropositive (N = 66)
**median age (1. and 3. quartile)**	30 (17–44)	71 (67–77)*
**mean household size (95%CI)**	5.2 (0–5.3)	5.0 (0–5.4)
**Mean number of sheep owned (95%CI)**	30.0 (25.8–34.2)	17.8 (12.8–22.7)
**sex**		
female	681 (68%)	47 (71%)
male	315 (32%)	19 (29%)
**living standard**		
good	481 (48%)	31 (47%)
middle	507 (51%)	33 (50%)
poor	8 (1%)	2 (3%)
**home slaughtering**		
no	88 (9%)	6 (9%)
yes	899 (91%)	60 (91%)
**cat ownership**		
no	714 (72%)	45 (68%)
yes	282 (28%)	21 (32%)
**cats in house**		
no	732 (73%)	49 (74%)
yes	252 (25%)	17 (26%)
**Water source**		
pipe	779 (78%)	53 (80%)
rivers	152 (15%)	11 (17%)
wells	51 (5%)	2 (3%)
**Consumption of Shashlyk**		
no	566 (61%)	35 (55%)
yes	356 (39%)	29 (45%)
**Consumption of home-made sour-cream**		
no	528 (57%)	35 (55%)
yes	395 (43%)	29 (45%)

* Significant difference between seropositive and seronegative participants (p<0.001).

**Table 3:** Results of mixed logistic-regression analysis of putative risk-factors for *T. gondii* seropositivity.

Risk factor	Estimate	Std. Error	z-value	p-value
**Age**	0.026621	0.007803	3.412	<0.001
**Poor living standard**	1.743616	0.848762	2.054	0.040
**Number of sheep owned**	−0.016161	0.008098	−1.996	0.045
**Consumption of Shashlyk**	0.494020	0.289153	1.709	0.088

### Model for the incidence of congenital toxoplasmosis in Kyrgyzstan

In 2008, 127,332 live births were recorded in Kyrgyzstan (latest available figures). Assuming that the toxoplasmosis prevalences in women from Bishkek and Kochkor measured in this study are representative of both urban and rural districts, we calculate that in 2008 approximately 175 (95% CI 136–216) children were born to mothers who seroconverted during pregnancy. This results in approximately 15 cases of chorioretinitis (7 present at birth, 8 with later onset), 6 intracranial calcifications, 1 hydrocephalus, 2 other CNS abnormalities and 2 neonatal or late foetal deaths.

### Model for the incidence of AIDS-related toxoplasmosis

Of a total of 3434 HIV-positive patients according to official government figures, 342 are estimated to be co-infected with *T. gondii*, of which 127 are at risk to develop AIDS related toxoplasmosis in the Kyrgyz Republic within the next 10 years. According to the estimate UNAIDS [9] the same numbers would amount to 9’700, 967 and 357, respectively. Estimated cases for the major two cities of Bishkek and Osh, as well as for the different oblasts are shown in Supporting information table S1.

## Discussion

The seroprevalence of *T. gondii* infection of 5.1% in the rural area of the Kyrgyz Republic was low compared to international numbers and to data from surrounding countries, whilst the seroprevalence in the Bishkek population (16.4%) is more in line with international data [6]–[8]. Several factors might contribute to the low seroprevalence in the rural study-population. Although consumption of undercooked meat and consumption of unwashed fruit or vegetables were associated with a higher infection risk in different studies [11], [12], [13], these are consumed only infrequently in rural Kyrgyzstan. Shashlyk (explained in the “Methods”-section) is the only meat dish eaten occasionally without thorough cooking, but this was not found to be a significant risk-factor. In the urban population of Bishkek, the type of food consumed is more likely to be a source of infection, especially as it frequently includes shashlyk and other undercooked meats, as well as salads, raw vegetables, and fruit which could be contaminated by oocysts. The risk for infection by oocysts is highlighted by several reported outbreaks of toxoplasmosis caused by contamination of surface water or public water supplies in various parts of the world [7]. Contaminated public water supplies may be a potential source of infection. However, in Bishkek the water supply is obtained mainly from underground sources and is of good quality with little treatment needed to make it potable. Likewise, in the rural study population, most of the water supplies (78%) came from pipes outside the house which are mainly fed by ground-water, where a low probability of contamination with oocysts of *T. gondii* can be assumed. To a minor degree these pipes are fed by reservoirs containing only spring water, where contamination with oocysts cannot be ruled out. Only a minority of the population (15%) takes drinking water from rivers. This was found to be a significant risk factor for toxoplasmosis in a similar region in Kazakhstan [5].

As there are no significant deviations in the linear increase of the odds ratio of seroprevalence with age, there is no evidence from the present data that the socioeconomic changes which began some 20 years ago have had an effect on the incidence of *T. gondii* infection.

Age, low social status and a low number of sheep owned were the only significant risk-factors in the rural population. Indeed, social status in this area is linked to a low number of sheep owned. On the other hand, people with high social status, income or education (e.g. medical personnel, teachers) in our study group own no or only few sheep which suggests that social status as well as low number of sheep are independent risk factors. In this respect, the results of our study are similar to those of a study performed in Kazakhstan, where a low number of cattle per family was also found to be a significant risk factor [5]. Different eating habits, especially consumption of vegetables, or differences in personal and food hygiene (e.g. hand-washing, washing of leaf vegetables) may be responsible for the higher prevalence in people with lower social status. A low education level, commonly linked to lower social status, has been found being a risk-factor for *T. gondii* infection [14], [15]. Home-slaughtering of sheep including direct contact with raw meat [13], [16] or consumption of unpasteurized milk [11], [13] were not significant risk factors. However, home-slaughtering is practised in nearly all households (91%), thus this factor was not discriminatory between affected and non-affected individuals. Other previously described, possible risk-factors are contact with soil [13], [17], [18], and poor personal and/or food hygiene [11], [19]. Since *T. gondii* oocysts are likely spread all over the settlements as cats roam freely, direct faecal-oral infection without food or water as an intermediate may be an important infection route. However, this is hard to document and evaluate, as almost all of the screened people work in agriculture and/or have regular contact with soil. The seroprevalence increased with age of participants consistent with other studies performed in various parts of the world (e.g. Europe, USA, Brazil, Kazakhstan [5], [9], [11], [14]).

The present results of our estimate on congenital toxoplasmosis indicate the possible extent of the problem. There are of course many assumptions in these calculations, the most important are that infection pressure has not changed over time, it is constant throughout the urban and rural regions of Kyrgyzstan and the sampled population is representative. The logistic regression approach to estimate the seroprevalence at each age allows for a higher infection pressure in children (indirectly through having an intercept) but otherwise would assume a more constant infection pressure. There is clearly uncertainty about this. Nevertheless, the seroprevalence in the sampled rural population is low compared with virtually all other countries [20] and is lower than found in a similar study in rural Kazakhstan [5]. Therefore, it can be argued that the incidence of congenital toxoplasmosis is unlikely to be substantially lower, but could indeed be higher than the calculated incidence. There is an important corollary to this. If the infection pressure in the population is higher, a greater proportion of girls will seroconvert before they reach reproductive age and hence a smaller proportion of women will be at a higher risk of seroconverting during pregnancy. Thus, there will be non-linear effects of increasing population exposure to *T. gondii* in terms of numbers of congenitally infected children. A mathematical model has suggested that an infection pressure representing approximately 4% seroconversions per annum amongst sero-negative individuals would result in the highest risks of congenital toxoplasmosis, with approximately 67% of women being seropositive at age 27. This would result in approximately 1% of pregnancies being affected by toxoplasmosis [21]. Higher infection pressure resulting in exposure of girls before reproductive age and lower infection pressures with a lower risk of exposure both result in fewer congenitally infected infants. The necessity of secondary prophylaxis by serological screening for and treatment of prenatal infection has currently been challenged in Europe within the last years as for example Denmark has effectively stopped its national prenatal screening program [22]. In contrast, primary prophylaxis by means of hygiene and adaptation of eating habits are still believed to be of importance in preventing the disease, although hard scientific data is lacking [23].

According to our estimate approximately 350–970 HIV-patients are currently co-infected with *T. gondii* in Kyrgyzstan, and between 125–360 AIDS patients are at risk for becoming infected within the next 10 years [24]. Thus, AIDS-related toxoplasmosis may cause considerable mortality, estimated to range between 0–17% (mean: 13%) in resource-poor settings even under treatment [25]–[28]. In addition there is disease morbidity, AIDS-related orphans and economic costs. Due to major differences in HIV-prevalence in official reports and in UNAIDS estimates we calculated two separate estimates with both these numbers. For this we assumed that the *T. gondii* infection seroprevalence levels were representative for the rural population, and the population of the two major Kyrgyz cities of Bishkek and Osh.

We estimate 66 clinically relevant cases of congenital infection and 125–360 cases of AIDS-related toxoplasmosis annually in the Kyrgyz Republic. Therefore, measures for primary prevention of *T. gondii* infection during pregnancy, education of the medical personnel and provision of urgently needed adequate material for diagnosis and treatment are essential for tackling toxoplasmosis in the future.

### Limitations of the study

Although we believe that the present study includes a representative sample of a mainly rural community of pastoralists and the urban population in Bishkek, there are important agrarian populations of Kyrgyz, Uzbek and Tajik ethnicity in the Fergana valley area of Southern Kyrgyzstan, which may have a different seroprevalence. Thus, the seroprevalence found in our study may not be representative for the entire country. Thus, the case numbers of clinically relevant toxoplasmosis have to be seen as an estimate representing mainly pastoralist communities and larger cities. Nevertheless, the magnitude of future putative clinical cases of toxoplasmosis can be derived from these estimates.

The sampling strategy was also not random as there were some departures from the normal population profile in Kyrgyzstan. In particular there is an over representation of women in our sample. This might produce some bias with regards to the factors associated with toxoplasmosis seropositivity in the rural population. However, the sampling strategy should neither affect the estimates of the incidence of congenital toxoplasmosis nor the incidence of complications of HIV infection as these were calculated from the age specific prevalences. This study design in which people were invited to participate clearly resulted in a disproportionate number of women entering the study and this may be because many men were unavailable due to work. In the rural population there were similar numbers of boys and girls in the study under 10 years of age which would be consistent with this hypothesis. The urban samples were supplied by diagnostic laboratories and it is not clear why these samples were over represented by women.

An additional limitation is the assumption that infection pressure or exposure has not changed with time. For example older people may have had greater exposure when they were younger and this could inflate the increase in age as a risk and overestimate the numbers of infants born with congenital infections. However changes in infection pressure would be expected to give non-linear increases in the age stratified sero prevalence. We tested this hypothesis by using generalised additive models which should have detected any significant deviations in the linear increase in the log of the odds ratio. Whilst this does not prove that infection pressure has not changed over time it does provide additional evidence that our assumptions are valid. On the contrary, the migration of individuals from rural areas with a low infection pressure to the cities with a higher infection pressure might mean that that this cross sectional study has underestimated the infection pressure and hence under estimated the number of cases of congenital toxoplasmosis.

The diagnostic test we developed and used in this study may have resulted in a small underestimate of the prevalence of toxoplasmosis. The Platelia Toxo IgG assay against which we evaluated our test system has a reported sensitivity and specificity approaching 100% [8]. Our assay was positive in 49 of 50 samples positive with the commercial test. This would indicate a sensitivity of 98% (CIs 89.35–99.95%) assuming that the Platelia Toxo IgG assay is indeed such a perfect gold standard. Therefore it is possible we have underestimated the prevalence of toxoplasmosis in these populations by approximately 2%.

## Supporting Information

Supporting information S1 Serological test design and serological analysis.
(DOC)

Supporting information S2 Methods used to estimates of incidence of congenital toxoplasmosis.
(DOC)

Supporting information S3 Methods used to estimates the numbers of cases of AIDS-related toxoplasmosis.
(DOC)

Table S1 Estimate of *T. gondii* and HIV co-infection and putative cases of AIDS-related toxoplasmosis in the two major cities and urban oblasts of the Kyrgyz Republic according to official data or UNAIDS estimates [1].
(DOC)

Checklist S1 STROBE checklist.
(DOC)
