# Supplementary material for: Toxoplasma gondii Infection in Kyrgyzstan: Seroprevalence, Risk Factor Analysis, and Estimate of Congenital and AIDS-Related Toxoplasmosis
Source: PLoS Negl Trop Dis. 2013 Feb 7;7(2):e2043. doi: 10.1371/journal.pntd.0002043 (PMC3566989; doi:10.1371/journal.pntd.0002043)
Supplement: Supporting information S1 — Serological test design and serological analysis. (DOC) [file pntd.0002043.s001.doc]

**Supplementary material 1.**

**Serological test design and serological analysis**

For the preparation of the *Toxoplasma* antigen to be used for coating ELISA plates, a monolayer of human foreskin fibroblasts (ATCC-LGC Standards Partnership; CRL-1634) was infected with tachyzoites of *T. gondii* (Strain CH-Rab 3962 D8/94) with an MOI of 10. Non-invasive tachyzoites were washed away after 2 hours. Following an incubation of 48 hours at 37°C most host cells were lysed. Extracellular tachyzoites were then harvested and washed 3 times in cold PBS (centrifuge 10min., 1000 x *g*, at 4°C). The pellet containing 5x10E8 parasites was re-suspended in 1 ml lysis buffer (1.5% Triton X-100 (Sigma-Aldrich, St. Louis MO), 5 mM EDTA, 30 µl/ml protease inhibitor cocktail (Protease Inhibitor Cocktail Nr. 1, Calbiochem, San Diego CA), 15 µl/ml PMSF (Sigma-Aldrich, St. Louis MO) solved in PBS) and incubated for 10 Min. in ice. After centrifugation at 4°C for 10 Min at 14’000 g, the supernatant was collected. Protein concentration was measured using the method of Bradford (Bio-Rad Protein Assay, Bio-Rad Laboratories, Hercules CA) and was 2.07 mg/ml. The optimal concentration of the antigen for coating was evaluated in titration experiments and was 10µg/ml.

Anti-*Toxoplasma* antibodies in serum samples were detected by ELISA at a dilution of 1:200 using standard ELISA-procedures [1] and the antigen described above. Rabbit anti-human IgG antibodies specific for the C_H_2 domain of the human gamma chain (DAKO, A0089) coupled to alkaline phosphatase (Roche Diagnostics, 567 752) were used as detection antibodies. Optical density was measured at 405nm (OD405nm). Control sera (negative, weakly positive and strongly positive) were included in all test runs. 48 *T. gondii*-negative human sera as defined by the results of the Platelia Toxo IgG Kit (Bio-Rad Laboratories, Hercules CA) were used for cut-off determination and evaluation of the test performance. The cut-off was defined as the mean OD405nm +3 standard-deviations of 47 *T. gondii*-negative samples (one outlier was removed). To evaluate the test’s performance, 50 negative (0 IU) and 50 positive sera (7-280 IU), as defined by the results of the Platelia Toxo IgG Kit (Bio-Rad Laboratories, Hercules CA) were measured. 49/50 gave a positive results in both tests (Pearson-correlation coefficient 0.9, p<0.001), and 50/50 scored negative in both tests, respectively.

**Reference:**

1. Schweiger A, Grimm F, Tanner I, Müllhaupt B, Bertogg K, et al. (2012) Serological diagnosis of echinococcosis: the diagnostic potential of native antigens. Infection 40(2): 139 - 152.
