# Supplementary material for: Toxoplasma gondii Infection in Kyrgyzstan: Seroprevalence, Risk Factor Analysis, and Estimate of Congenital and AIDS-Related Toxoplasmosis
Source: PLoS Negl Trop Dis. 2013 Feb 7;7(2):e2043. doi: 10.1371/journal.pntd.0002043 (PMC3566989; doi:10.1371/journal.pntd.0002043)
Supplement: Supporting information S2 — Methods used to estimates of incidence of congenital toxoplasmosis. (DOC) [file pntd.0002043.s002.doc]

**Supplementary material 2**

**Methods used to estimates of incidence of congenital toxoplasmosis**

The variation of seropositivity was estimated using a logistic regression model. As age was a significant regressor, the model can be used to estimate the prevalence of seropositivity of any age group. The differences in the seropositivity of women of reproductive age t and t+0.75 years give an estimate of the proportion of pregnant women of age t who seroconvert during pregnancy. Confidence intervals were also calculated by using the likelihood profile of the fit to the logistic regression model.

The numbers of annual births for each age group of women in Kyrgyzstan was obtained from United Nations data bases ([http://data.un.org](http://data.un.org/)).

The probability for pregnancy in each age group was multiplied by the number of births to estimate the total number of births to women who seroconverted during pregnancy. Of those, only a minority is likely to give birth to a child with congenital toxoplasmosis [1]. According to Kortbeek et al. [2] approximately 13% of CT cases have chorioretinitis, 11% intracerebral calcifications, 2% hydrocephalus, 3% other CNS abnormalities, 0.7% suffer neonatal death and 2.8% suffer late foetal death (after 24 weeks). An additional 16% will suffer chorioretinitis later in life. A systematic review that included cohort data from 1721 women who seroconverted during pregnancy demonstrated that there is a 29% transmission rate to the foetuses [3].

**References:**

1. Bénard A, Salmi LR (2006) Systematic review of published data on the burden of congenital toxoplasmosis in Europe (Panel 1: burden of the disease issues). Eurotoxo.

2. Kortbeek LM, Hofhuis A, Nijhuis CD, Havelaar AH (2009) Congenital toxoplasmosis and DALYs in the Netherlands*.* Mem Inst Oswaldo Cruz 104(2): 370-373.

3. Thiebaut R, Leproust S, Chêne G, Gilbert R (for SYROCOT) (2007) Effectiveness of prenatal treatment for congenital toxoplasmosis: a meta-analysis of individual patients' data. Lancet 369(9556): 115-122.
