# Supplementary material for: Toxoplasma gondii Infection in Kyrgyzstan: Seroprevalence, Risk Factor Analysis, and Estimate of Congenital and AIDS-Related Toxoplasmosis
Source: PLoS Negl Trop Dis. 2013 Feb 7;7(2):e2043. doi: 10.1371/journal.pntd.0002043 (PMC3566989; doi:10.1371/journal.pntd.0002043)
Supplement: Supporting information S3 — Methods used to estimates the numbers of cases of AIDS-related toxoplasmosis. (DOC) [file pntd.0002043.s003.doc]

**Supplementary material 3.**

**Methods used to estimates the numbers of cases of AIDS-related toxoplasmosis**

The source for information on HIV-Patients in Kyrgyzstan was the national statistical office of the Kyrgyz Republic ([www.stat.kg](http://www.stat.kg/)), and the “UNAIDS: 2010 Report on the global AIDS epidemic” (available online: www.unaids.org/globalreport). The prevalence of HIV-infections was estimated at 0.02% (>0.02-0.04%) in 2001 and 0.2% (0.13-0.32) in 2009, respectively, adding up to 9’700 (6’400-16’000) HIV-patients in 2009. The calculated overage with highly active antiretroviral treatment (HAART) in this time period was only 12% [1, 2, 3]. The majority of HIV-patients will meet the criteria for HAART within the next 10 years based the normally observed progression of HIV-infection [4]. The known combined risk of AIDS-related toxoplasmosis varies between 31.5-52% in *T. gondii*-positive patients in need of HAART [, 5, 6, 7], thus a mean risk of 40% was used in our study. Due to variations in age-specific prevalences of HIV, and toxoplasmosis, respectively, age-specific data from our study and for HIV (age groups 0-4 (7%), 5-14 (1%), 15-17 (1%), 18-19 (2%), 20-24 (14%), 25-29 (23%), 30-34 (21%), 35-39 (16%), and ≥ 40 years (15%; source: National Centre for AIDS of the Kyrgyz Republic: ww.aids.gov.kg) were used in our calculations.

Thus the calculation of putative future AIDS- related toxoplasmosis cases was performed as follows:

Putative future cases = Σ (PHIV * PToxoplasma*Pop) * RAT * %PwoH

Where PHIV is the prevalence of HIV-patients and PToxoplasma the seroprevalence of Toxoplasmosis in the defined age and population groups, Pop is the population at risk, RAT the risk of AIDS related toxoplasmosis in co-infected patients and % PwoH the HAART-coverage according to UNAIDS.

**References**

1. Brown T, Bao L, Raftery AE, Salomon JA, Baggaley RF, et al. (2009) Modelling HIV epidemics in the antiretroviral era: the UNAIDS Estimation and Projection package. Sex Transm Infect 86 Suppl 2: ii3-10.

2. Bao L, Raftery AE (2010) A stochastic infection rate model for estimating and projecting national HIV prevalence rate*s.* Sex Transm Infect 86 Suppl 2: ii93-99.

3. Stanecki K, Daher J, Stover J, Beusenberg M, Souteyrand Y, et al. (2010) Antiretroviral therapy needs: the effect of changing global guidelines. Sex Transm Infect 86 Suppl 2: ii62-66.

4. Vergis EN, Mellors JW (2000) Natural history of HIV-1 infection. Infect Dis Clin North Am 14(4): 809-825.

5. Luft BJ, Remington JS (1992) Toxoplasmic encephalitis in AIDS*.* Clin Infect Dis 15(2): 211-222.

6. Dannemann B, McCutchan JA, Israelski D, Antoniskis D, Leport C, et al. (1992) Treatment of toxoplasmic encephalitis in patients with AIDS. A randomized trial comparing pyrimethamine plus clindamycin to pyrimethamine plus sulfadiazine. The California Collaborative Treatment Group. Ann Intern Med 116(1): 33-43.

7. Rabaud C, May T, Amiel C, Katlama C, Leport C, et al. (1994) Extracerebral toxoplasmosis in patients infected with HIV. A French National Survey. Medicine (Baltimore**)** 73(6): 306-314.
