# Supplementary material for: Toxoplasma gondii Infection in Kyrgyzstan: Seroprevalence, Risk Factor Analysis, and Estimate of Congenital and AIDS-Related Toxoplasmosis
Source: PLoS Negl Trop Dis. 2013 Feb 7;7(2):e2043. doi: 10.1371/journal.pntd.0002043 (PMC3566989; doi:10.1371/journal.pntd.0002043)
Supplement: Table S1 — Estimate of T. gondii and HIV co-infection and putative cases of AIDS-related toxoplasmosis in the two major cities and urban oblasts of the Kyrgyz Republic according to official data or UNAIDS estimates [1]. (DOC) [file pntd.0002043.s004.doc]

**Supporting information table S1:** Estimate of *T. gondii* and HIV co-infection and putative cases of AIDS-related toxoplasmosis in the two major cities and urban oblasts of the Kyrgyz Republic according to official data or UNAIDS estimates [1].

|  | **Case numbers (95%CI)** |  | | |
| --- | --- | --- | --- | --- |
|  | **Official data** | **UNAIDS estimate** | | |
| **Location** | **Population** | **HIV -positive** | ***T. gondii*** **-HIV-co-infection** | **AIDS related toxoplasmosis** |
| **Major cities** |  |  |  |  |
| Bishkek | 865'527 | 519 | 90 (73-109) | 33 (24-46) |
| Osh | 243'216 | 727 | 126 (105-149) | 46 (35-61) |
| **Total urban** | **1'108'743** | **1246** | **216 (188-246)** | **80 (63-98)** |
| **Rural Oblasts** |  |  |  |  |
| Chuy | 818'000 | 1001 | 58 (45-75) | 21 (14-32) |
| Osh (w/o City) | 1'056'284 | 658 | 38 (28-52) | 14 (8-24) |
| Jalal-Abad | 962'200 | 364 | 21 (14-32) | 8 (4-15) |
| Batken | 400'400 | 56 | 3 (1-9) | 1 (0-6) |
| Issyk-Kul | 450'700 | 53 | 3 (1-9) | 1 (0-6) |
| Naryn | 269'700 | 30 | 2 (0-5) | 1 (0-4) |
| Talas | 216'100 | 26 | 2 (0-6) | 1 (0-4) |
| **Total rural** | **4'173'384** | **2188** | **127 (105-149)** | **47 (34-61)** |
| **Whole country** | **5'282'127** | **3434 (65.0)** | **342 (306-380)** | **127 (105-149)** |

**References**

1. UNAIDS (2010) Global report: UNAIDS report on the global AIDS epidemic 2010. Geneva: UNAIDS.

359 p.
